# Regulating duty free sales and tobacco advertising in airports: a call for action

**DOI:** 10.1186/1617-9625-9-7

**Published:** 2011-06-23

**Authors:** Constantine I Vardavas, Andrew B Seidenberg, Gregory N Connolly

**Affiliations:** 1 Center for Global Tobacco Control, Department of Society, Human Development and Health, Harvard School of Public Health, (677 Huntington Avenue), Boston, MA (02115), USA

## 

According to the United Nations' World Tourism Organization, there were an estimated 880 million international travelers in 2009, or roughly 13% of the world's population [1,2]. While tobacco remains one of the world's leading causes of preventable disease and death, prompting numerous governments around the world to implement policies to protect the public's health, many international airports remain exempt from tobacco control restrictions. Tobacco advertising and tobacco sales are largely unregulated in many international airports, exposing hundreds of millions of travelers each year to the tobacco industry's influence. Consequently, airports may represent one of the last remaining havens for the tobacco industry.

National and international tobacco regulations (such as article 13 of the FCTC) have been promoted to prevent the tobacco industry from communicating with youth and other susceptible populations [3]. Despite the existence of such regulations, indoor tobacco billboards (Figure 1, Figure 2), industry-sponsored smoking rooms (Figure 3, Figure 4), and point of purchase advertising (Figure 5, Figure 6) are found within airports throughout the world, including those located within jurisdictions with tobacco advertising restrictions in place.

**Figure 1:**
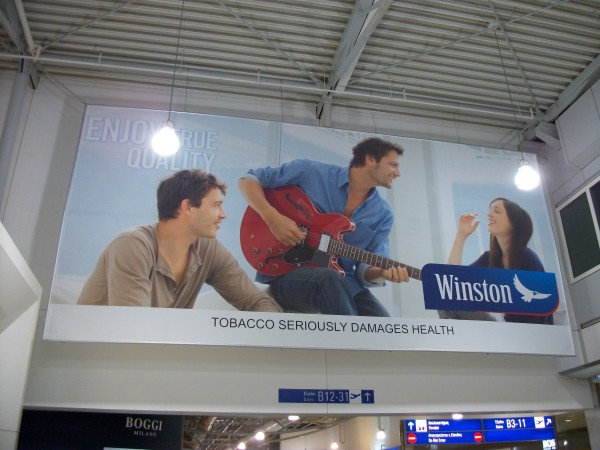
**Indoor billboard advertising after passport control in Athens airport**.

**Figure 2:**
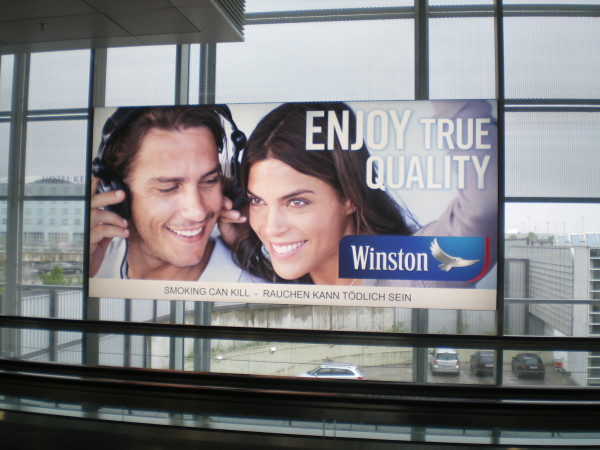
**Indoor billboard advertising between boarding gates in Munich airport**.

**Figure 3:**
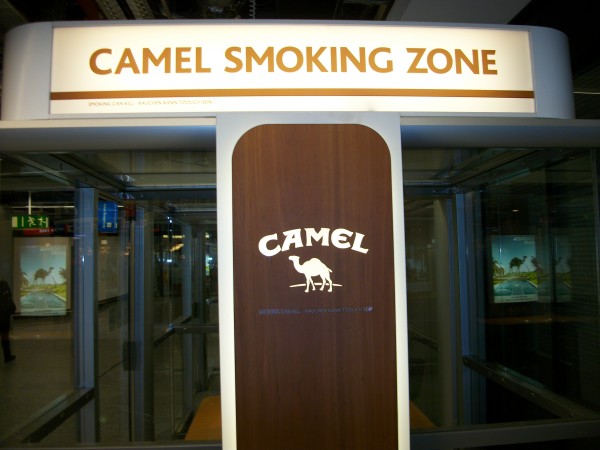
**Brand advertising (Camel) on a smoking room in Frankfurt airport**.

**Figure 4:**
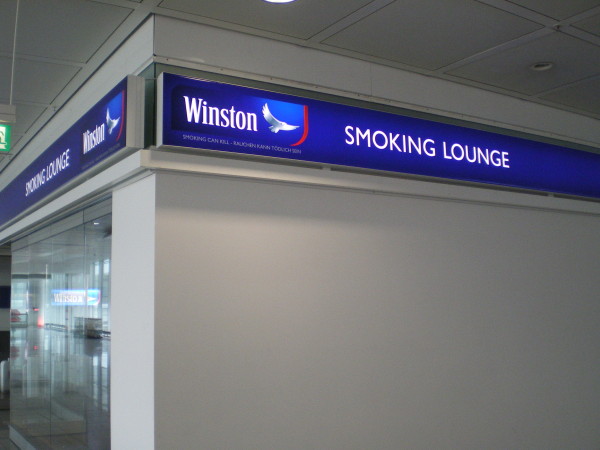
**Brand advertising (Winston) on a smoking room in Munich airport**.

**Figure 5:**
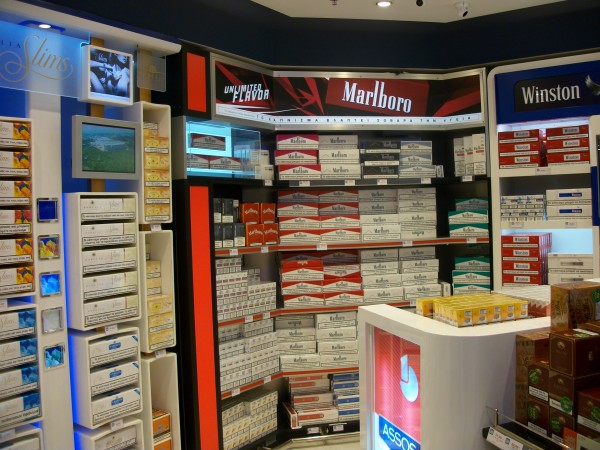
**Point of purchase advertising with the use of colors brand names and television screens in Athens airport**.

**Figure 6:**
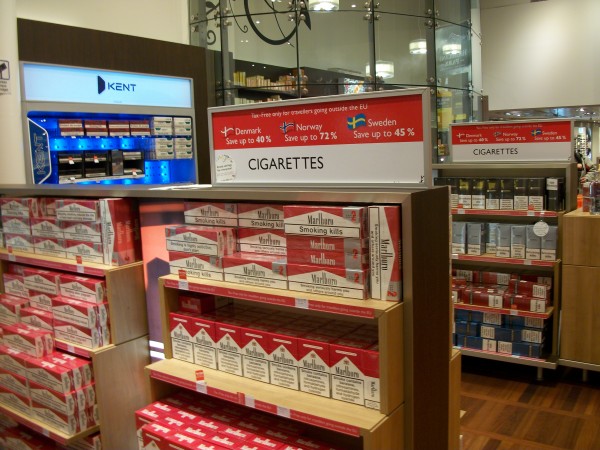
**Point of purchase advertising, stressing duty free prices, in Copenhagen airport**.

Presented in vivid, colorful displays portraying brand names, themes, and logos (Figure 7), and often promoted by both salepersons and through promotional paraphernalia, airport point of sale advertising may stimulate tobacco purchases. Indeed, research has indicated that point of sale advertising has an impact on impulse purchases among current smokers, and recent quitters, with those more sensitive to such advertising less likely to remain abstinent [4-6]. When combined with the reduced price of tobacco products within airports, one can conceive the synergistic effect of reduced price and plethoric advertising which could facilitate impulse purchases, smoking relapse and circumvent one of the key aspects of tobacco control, which is taxation and product price regulation [7].

**Figure 7:**
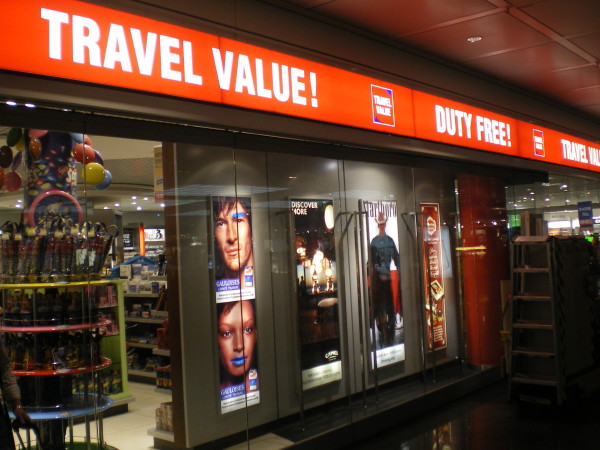
**Brand advertising within Munich airport duty free sales areas**.

Duty free sales undermine the purpose of taxation and harms public health by encouraging personal consumption. Research from a number of countries has indicated that the percentage of smokers consuming tobacco from a duty free source ranges from below 1% in the US, Canada and Australia, and up to 3.8% and 5.6% in New Zealand and the UK, respectively [7-9]. When one extrapolates the number of cigarettes smoked per capita per day from duty free sources to a global level one can grasp a hold of the loss of tax revenue that could be provided to support tobacco control efforts or population based health care coverage and thus the subsequent implications on global public health.

The time has come to discuss the rational behind exemptions for airport advertising and duty free tobacco sales. Advertising and purchases within international travel should be governed by universal regulations such as those promoted by the FCTC and action should be taken towards their implementation at a global level as the tobacco industry does not recognize borders or boundaries [3,10].

*Tobacco Induced Diseases*****embraces the need for the collection of basic research on tobacco promotion and advertising within airports as a means for promoting evidence based advocacy.

## Conflict of interest

The authors declare that they have no competing interests.
